# Trained Immunity as a Prospective Tool against Emerging Respiratory Pathogens

**DOI:** 10.3390/vaccines10111932

**Published:** 2022-11-15

**Authors:** John Joseph

**Affiliations:** Center for Nanomedicine, Department of Anesthesiology, Perioperative and Pain Medicine, Brigham and Women’s Hospital, Harvard Medical School, Boston, MA 02115, USA; jjoseph39@bwh.harvard.edu

**Keywords:** trained immunity, innate immune memory, respiratory pathogens, BCG, next-generation vaccines, COVID-19

## Abstract

Although parental vaccines offer long-term protection against homologous strains, they rely exclusively on adaptive immune memory to produce neutralizing antibodies that are ineffective against emerging viral variants. Growing evidence highlights the multifaceted functions of trained immunity to elicit a rapid and enhanced innate response against unrelated stimuli or pathogens to subsequent triggers. This review discusses the protective role of trained immunity against respiratory pathogens and the experimental models essential for evaluating novel inducers of trained immunity. The review further elaborates on the potential of trained immunity to leverage protection against pathogens via the molecular patterns of antigens by pathogen recognition receptors (PPRs) on innate immune cells. The review also focuses on integrating trained innate memory with adaptive memory to shape next-generation vaccines by coupling each one’s unique characteristics.

## 1. Introduction

Innate immunity elicits a nonspecific defense against the entry of respiratory pathogens, as modulated by neutrophils, monocytes, macrophages, dendritic, natural killer (NK) cells, and gamma delta (γδ) T cells [1,2,3,4,5,6]. A timely and robust innate immune response can impede the invasion of respiratory pathogens to attenuate disease symptoms or severity [7]. Despite recent advances in vaccine technology, the COVID-19 pandemic exposed the vulnerability of vaccines against evolving SARS-CoV-2 variants due to their dependency on antigen-specific, antibody-mediated response [8]. Recent compelling evidence highlights the role of innate immune memory in the induction of a more robust, nonspecific response against unrelated pathogens following exposure to certain immunostimulants (or after a microbial infection), defined as ‘trained immunity’ or ‘innate immune memory.’ This early immune response confers long-term protection against unrelated pathogenic threats for several months to years [9,10,11,12]. Training immunity is pertinent in the advent of unprecedented respiratory infections, which are usually limited to the timeline of vaccine development. This review highlights the role of trained immunity against respiratory pathogens such as SARS-CoV-2, influenza, RSV (Respiratory Syncytial Virus), *Staphylococcus aureus*, *Escherichia coli*, and *Streptococcus pneumoniae*. Although numerous reviews on innate immune memory detail the mechanism of trained immunity, their inducers, and the role of effector immune cells, for the first time, this review spotlights the experimental models of trained immunity and the recent advancements to circumvent the growing demand for broad-spectrum protection against respiratory pathogens. In this context, the review emphasizes the integration of trained immunity inducers with antigens to confer the traits of innate and adaptive immune memory to produce next-generation vaccines. This hybrid vaccination approach could be leveraged to address the unmet need to develop fast and efficient immunization strategies and holds tremendous potential to create a paradigm shift in prophylaxis against emerging pathogens.

## 2. Characteristics of Adaptive and Trained Immunity

Classical vaccines mediate long-lasting adaptive immunity from antigen-specific B and T memory cells. Although vaccines are highly effective against the pathogen strain against which it was originally developed, they are considered less efficient against variants. However, certain vaccines such as Bacillus Calmette–Guérin (BCG, an attenuated bacterial vaccine derived from *Mycobacterium bovis*), influenza, polio, or measles-mumps-rubella (MMR) bestow heterogenous protection by the activation of trained innate immunity [13,14,15,16]. The non-specific recognition of pathogens occurs through the diverse pathogen recognition receptors (PPRs) [17]. The trained immune response is manifested by metabolic reprogramming and epigenetic modifications happening at the transcriptional level compared to the gene recombination and clonal expansion, a hallmark feature of adaptive immunity [18,19]. The onset of innate immune memory upon a secondary challenge (exposure to pathogens or antigens) is faster and typically takes minutes to hours, resulting in elevated non-specific innate response mediated by the secretion of higher levels of proinflammatory cytokines, reactive oxygen species (ROS), and phagocytosis [20].

Chromatin is a repeated unit of nucleosome that comprises 147 bp of DNA wrapped around in octameric histone proteins with a pair of H3 and H4 homodimers and a set of H2A/H2B heterodimers [21]. Methylation and acetylation occur at the N-terminal histone tails induced by enzymes, such as histone acetyltransferase (HAT), histone deacetylase (HDAC), lysine methyltransferase (KMT), and lysine demethylase (KDM). Gene expression is regulated by the extent of histone modification facilitating the accessibility of transcriptional machinery to DNA. A marked transcriptional change occurs with H3 histone as exemplified by HAT-mediated acetylation at lysine 9 (H3K9ac) and lysine 27 (H3K27ac), which are associated with elevated inflammation in innate immune cells [22,23]. On the contrary, HADC enzymes facilitate the removal of an acetyl group from the histone tail. A balance in HAT/HDAC is an essential factor determining the gene expression [24]. Similarly, KMT and KDM enzymes are highly specific to amino acids in the H3 histones. Alterations in the location of a lysine residue in histones determine the functional outcome. A repressed gene is correlated with the methylation of lysine9 (H3K9me) and lysine27(H3K27me) at H3 histone. In striking contrast, a permissive epigenetic modification is characterized by the methylation of lysine4 (H3K4me) and lysine36 (H3K36me) [25]. Moreover, transcriptional activity depends on the degree of methylation, which can be mono, di, or trimethylation on H3 histone. For instance, transcription was more profound for trimethylated H3 histone in lysine4 (H3K4m3) at gene promoters [26]. These epigenetic modifications by acetylation and methylation of histone confer gene transcription that elicits an antipathogen response after the primary exposure to a trained immunity stimulant [25,27].

**Figure 1:**
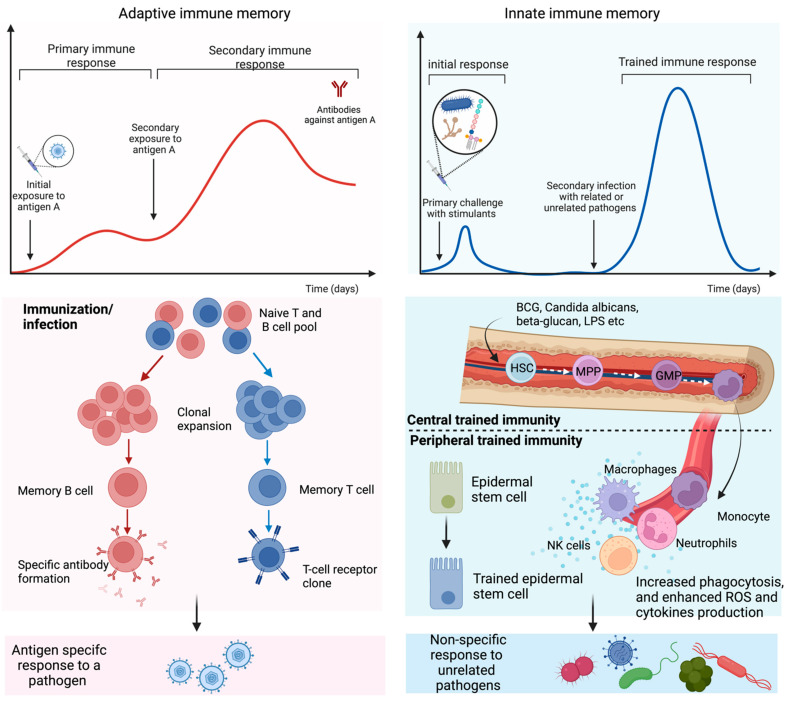
Adaptive and trained immune response. Exposure to antigens or pathogens may trigger innate and adaptive immune responses. In adaptive immunity, an antigen-specific response is attributed to antibody production and long-lived B and T lymphocytes by gene recombination and clonal expansion. Trained immunity is governed by a rapid and heightened innate immune response to certain vaccines and antigens that reprogram the hematopoietic stem cells (HSC) residing in the bone marrow or circulating innate immune cells. HSC undergoes differentiation to bone marrow precursors, multipotent progenitor (MPP), and granulocyte-macrophage progenitor (GMP) and consequently enters peripheral organs as circulating monocytes and tissue macrophages. Epithelial stem cells have also been identified as part of the peripheral trained immunity along with dendritic, NK, and endothelial cells.

In addition, the primary challenge of immunostimulants after recognizing through PRRs prompts a cascade of intracellular metabolic pathways in immune cells, such as the tricarboxylic acid (TCA) cycle, glycolysis, and fatty acid metabolism. Metabolites involved in these processes, such as acetyl coenzyme A (acetyl-CoA) and fumarate, may also facilitate epigenetic reprogramming of the cells through the activation or inhibition of enzymes [18]. These reprogramming of the molecular events occur at long-lived hematopoietic stem cells (HSC) in the bone marrow (centrally trained immunity) and short-lived circulating immune cells, including monocytes, macrophages, natural killer (NK) cells, etc. (Figure 1). It is comprehended that HSC-derived monocytes differentiate into macrophages with augmented effector functions in peripheral regions. Similarly, NK cells with innate immune memory provide enhanced antipathogenic response by faster degranulation and cytokine release than NKs in the normal state. Respiratory epithelial stem cells also featured characteristic innate immune memory by altering chromatin accessibility upon exposure to the stimulus [28].

Several studies reported the reprogramming of myeloid cells following exposure to microbes such as fungi and viruses [29,30,31]. Growing evidence of literature indicates the immune memory of dendritic cells (DCs) upon exposure to the fungus, *Cryptococcus neoformans* in the murine model [3,32,33]. On a subsequent challenge with the same pathogen, epigenetic modifications were observed to trigger the robust production of interferon γ (IFNγ) and Th1 cytokines. This phenomenon was impeded upon treating the animals with histone methyltransferase inhibitors. In addition, herpes infection enhanced the protection against *Yersinia pestis* via IFNγ and activation of circulating macrophages [34,35]. Another study revealed the characteristic memory similar to adaptive immunity in NK cells when mice were exposed to mouse or human cytomegalovirus [36,37,38].

Similarly, vaccines and vaccine adjuvants have been associated with triggering innate immune memory against early pathogen invasion. For instance, exposure of β-glucan, a polysaccharide of the fungal cell wall, or *C. albicans* to macrophages or monocytes elicited a profound response in subsequent challenges to related or PAMPs or microorganisms, including parasites and viruses [39]. Key inducers, including IL-1β and GM-CSF (granulocyte-macrophage colony-stimulating factor), were necessary for β-glucan-mediated trained immunity [40,41]. Intriguingly, Toll-like receptors or NOD-like receptors agonists were effective against *Toxoplasma* and *E. coli*, respectively [42,43,44]. Likewise, compelling evidence of BCG vaccines unveiled a profound effect on preventing infection without involving adaptive immune cells [15,45]. The nonspecific innate memory induced by BCG was mediated by type II interferon. BCG also induced heterogeneous protection resulting in low parasitemia and viremia in the clinical trial with malarial infection and yellow fever, respectively [46,47]. Most importantly, prophylaxis was not exclusively against tuberculosis (TB) but against several respiratory infections, as detailed in the upcoming section.

Contrary to trained immunity, ‘tolerance’ is characterized by a change in the programming of the immune cells, when subjected to persistent exposure to stimulants, leading to a slower response and gene transcription [40]. This change in the adaptive programming state of the cells is decisive on factors such as the duration (long or short) of stimulation and the dose (low or high) of stimulants [40].

## 3. Cross-Protection of Trained Immunity against Respiratory Infections

The recent decades witnessed the increased incidence of epidemics and pandemics caused by respiratory tract infections imposing a significant socio-economic burden [48,49]. A growing body of evidence suggests the therapeutic efficacy of trained immunity to confer prophylaxis against respiratory infections caused by bacteria and viruses (Table 1). Harnessing trained immunity could be leveraged as an effective prophylactic tool to bolster the innate immune system to defend against pathogens.

The emergent variants of the influenza virus led to pandemics and seasonal epidemics worldwide, affecting millions of lives. In a randomized pilot study, the administration of BCG vaccines before intramuscular influenza vaccination in healthy volunteers augmented the production of antibody response against the H1N1pdm09 influenza virus [50]. The hemagglutination-inhibiting antibody titer was remarkably higher in the BCG vaccinated cohort than in the placebo group. In another study, intranasal delivery of BCG in murine models enhanced efferocytosis by alveolar phagocytes and maintained lung homeostasis against a lethal mouse-adapted influenza virus A/Puerto Rico/8/34 (PR8) (H1N1) strain [51]. Subcutaneous immunization of BCG failed to confer protection from PR8 infections, and 100% of the animals succumbed after 15 days post-infection. This study connotes the relevance of the administration route of BCG to function as an inducer of trained innate immunity. Besides BCG, other inducers also presented evidence to ponder the potential of trained immunity against the influenza virus. Extensive clinical trials on BCG-induced trained immunity for suppressing disease severity and pathogenesis of SARS-CoV-2 will proffer convincing evidence to bridge the gap for early prophylaxis until an antigen-specific vaccine becomes available. The non-targeted protection against acute lung infections caused by respiratory syncytial viruses in newborns further complements the ubiquitous induction of trained immunity by BCG vaccines [52].

Brandi et al. evaluated the degree of protection of an inactivated poly-bacterial mucosal immunomodulator, MV130 [53]. Intranasal administration of this immune inducer confers heterologous protection against antigenically unrelated respiratory pathogens such as vaccinia and influenza A/Puerto Rico/8/1934 (H1N1) (PR8) viruses. The study further elucidated the mechanistic role of MV130 with comprehensive landscaping of lung immune cells. The prophylaxis of MV130 induces a transient invasion of CD4+ and CD8+ T cells, alveolar macrophages, and neutrophils to the lungs. In addition, the study validated the association of trained immunity as an attributing factor to the defensive role of MV130 with a counter-treatment of an mTOR inhibitor that blocks the induction of trained immunity. The efficacy of MV130 in inducing heterologous protection against SARS-CoV-2 was evaluated in K18-hACE2 mice [54]. The potency of MV130 to elicit cellular immunity was also demonstrated ex vivo by immunomodulating human dendritic cells to promote Th17 and IL-10-producing T cells [55].

Intranasal exposure of lipopolysaccharides (LPS) exhibits a noticeable immunogenic activity upon challenge with *S. pneumoniae* and rewires the metabolism of alveolar macrophages to abrogate the infection [56]. Other adjuvants such as zymosan also orchestrate trained immunity to confer broad-spectrum protection against peritonitis and pneumonia caused by a wide array of pathogens such as *S. aureus*, *E. coli*, and *Pseudomonas aeruginosa* [43]. Together, these research efforts underline the potency of trained immunity inducers to leverage non-specific innate responses against heterologous respiratory pathogens. However, preclinical and clinical studies need to address the longevity of the protection, frequency of administration, and ubiquitous use for all populations, including immunosenescent, immunocompromised, and infants.

**Table 1:** Cross-reactivity of trained immunity induced by various stimulants against respiratory pathogens.

Stimulation	Model	Cell Type	Cross Protection	Remarks
BCG (i.n)	Mouse	Monocytes	Influenza A	Intranasal administration of BCG resulted in 100% survival against PR-8 [51]
BCG	Ex vivo- Human neutrophils	Neutrophils	NA	Increased IL-1β, IL-8, ROS production, Phagocytosis [57]
BCG (i.m)	Human and Ex vivo	NA	Influenza A	BCG modulate innate immune response. Influenza vaccines augment acquired immunity at higher levels [50]
BCG (i.d)	Humans (neonates)	NA	NA	Reduced infectious disease related mortality by 43% [58]
BCG	Humans (neonates)	NA	RSV	Reduced risk of acute lower respiratory tract infection [52]
BCG (i.d)	Humans (elderly)	NA	NA	Reduce disease severity by attenuating matrix metalloproteinases (MMPs), and pro-inflammatory cytokines [59]
MV130 (i.n)	Mouse	NA	SARS-CoV-2	Improved the survival and elevated B and T lymphocyte response [54]
MV130	Ex vivo and mouse	Dendritic cells	NA	Increased the level of TNF-α, IL-6, IL-1β, and IL-23 [55]
MV130	Ex vivo and mouse	Monocytes	Influenza A	Improved survival [53]
β-glucan	Mouse	Monocytes	*C. albicans*	Enhanced TNF-α and IL-6 production. Improved survival against *C. albicans* [4]
LPS	Ex vivo and human	Monocytes	Influenza A	Enhanced production of TNF-α, IFN-β, and IFN-γ [56]

i.n—intranasal, i.m—intramuscular, i.d—intradermal.

## 4. Experimental Design and Models of Trained Immunity

**Figure 2:**
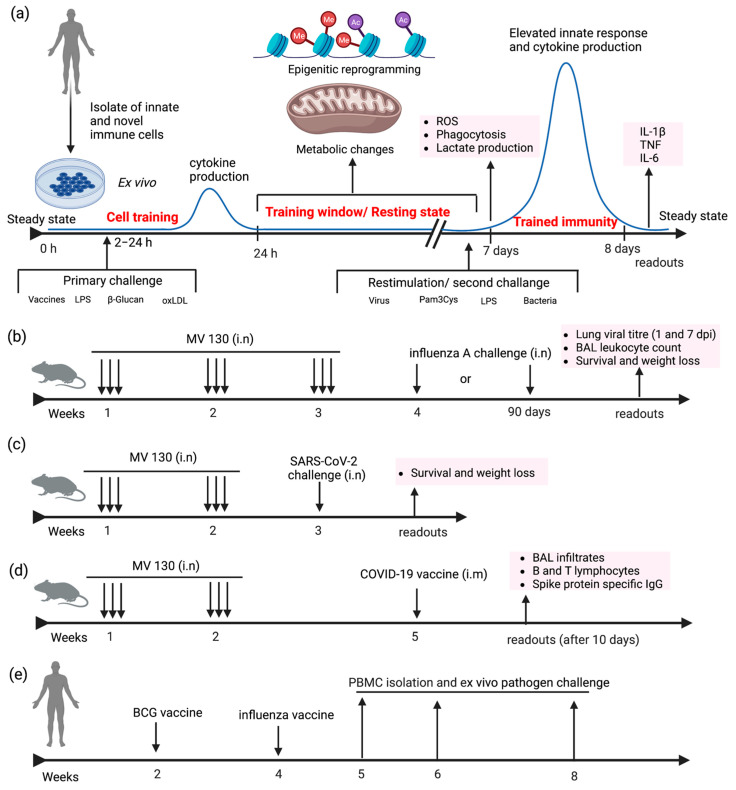
Examples of diverse experimental designs and models of trained immunity. (**a**) Ex vivo stimulation of innate immune cells isolated from humans. Cells are stimulated with vaccines or adjuvants, and cytokine levels are quantified after 24 h. Culture media is replaced to remove the antigens, and cells undergo epigenetic and metabolic modification. After 5–7 days of the resting phase, cells produce heightened innate responses following a re-exposure to old/new stimuli. (**b**–**d**) Murine model to evaluate the immunological effect of MV130 against influenza A, SARS-CoV-2, or COVID-19 vaccines [53,54] (**e**) Human model to investigate the effect of BCG on the influenza vaccine [60].

A predominant in vitro/ex vivo model to study trained immunity involves the treatment of human peripheral monocytes with stimulants for a brief duration ranging from 2–24 h (Figure 2a). The stimulus is withdrawn, and the cell returns to a functional, steady state. This resting period is maintained for 24 h or 3–7 days, where the cells undergo epigenetic and metabolic rewiring, which will lead to an enhanced response to homologous or heterologous pathogens or stimuli [60]. Restimulation was performed with influenza, Pam3Cys, or LPS for 1–24 h. The trained cells on secondary challenge upregulate the production of ROS and cytokines and augment cell death by phagocytosis [60]. In vivo model provides insight into the reprogramming of HSCs residing in the bone marrow along with the changes in innate cells in the peripheral organs. Stimulants like BCG vaccines and β-glucan result in the proliferation and expansion of HSCs as part of the centrally trained immunity. Mouse models have been extensively used to evaluate the outcome of central and peripheral trained immunity against homologous and heterologous pathogens. In recent work, C57BL/6 mice was used to evaluate the trained immune response of intranasally administered MV130 [53]. Animals were pretreated with MV130 three times per week for two weeks, followed by the pathogen challenge either with influenza or *C. albicans* after a resting phase of one week (Figure 2b). Animal body weight and survival were monitored for two weeks after the resting phase. Lung viral titer was enumerated on days 1 and 7 after the last administration of MV130. The longevity of protection was also monitored three months after the administration of MV130. Mouse bone marrow progenitor cells were analyzed for epigenetic modification. In another report, K18-hACE2 mice models were used to evaluate the protection against SARS-CoV-2 infection following pre-treatment intranasal administration of MV130 thrice weekly for two weeks. After a resting period of one week, animals were intranasally infected with 10^4^ PFU (Plaque Forming Units) of SARS-CoV-2 (MAD6 strain) (Figure 2c). Body weight and survival were monitored for 14 days [54]. On another note, C57BL/6 mice were used to determine the effect of MV130 on augmenting the immunogenicity of two different COVID-19 vaccines. MV130 and MVA-CoV2-S (modified vaccinia virus Ankara (MVA) vector expressing SARS-CoV-2 spike (S) protein (MVA-S)) vaccines were administered at three weeks intervals, and myeloid and lymphoid immune populations were analyzed after ten days (Figure 2d).

Numerous clinical trials have been conducted in neonates [61], adults, and the elderly population [62] to investigate the effect of BCG on trained immunity. The reprogramming of HSCs and peripheral monocytes following BCG vaccination was established in human models. Ex vivo challenge experiments were performed after one month [4] to bestow the clearance of stimulants. The secondary challenge can be pathogens or stimulants such as LPS, Pam3Cys, etc. Clinical trials were also conducted to evaluate the immunological effect of BCG vaccines in the COVID-19 hotspot area [50,57]. Blood plasma evaluations of MMPs, chemokines, and cytokines were performed one month after the vaccination (Figure 2e).

## 5. Potential of Trained Immunity to Fight Emerging Pathogens

The recent epidemics and pandemics associated with emerging respiratory viruses have exposed vulnerabilities in tedious, traditional vaccine development and the suboptimal efficacy of vaccines against heterogeneous strains, owing to antigen-specific IgG responses. Although nasal vaccines show elevated levels of mucosal protection compared to systemic vaccines, regulatory hurdles and a lack of appropriate mucosal adjuvants have decelerated their development [63,64]. Despite years of research, FluMist® (Astra Zeneca, Cambridge, UK) remains the only FDA-approved nasal vaccine, which unfortunately showed inconsistent efficacy in heterogeneous patient populations [65]. Our review highlights the pivotal role of inducers of trained immunity, especially BCG, in preventing pathogen invasion, decreased viral load, disease severity, and mortality in preclinical and multicentric clinical trials. Immunodeficiency, genetic predisposition, comorbidities, age, etc. are all attributes that weaken the innate antiviral response and increase exposure to respiratory infection [66]. Moreover, recent studies spotlight the protective role of BCG’s trained immunity in reducing the severity of SARS-CoV-2 infection in the elderly [59,62]. BCG-induced trained immunity also synergistically bolstered cytokine induction of influenza vaccines, and subsequent protection, against respiratory viruses [50]. In these contexts, I envision that trained immunity may represent a complementary prophylactic tool to evoke a timely and robust innate immune response before the availability of antigen-specific vaccines. Thus, inducers of trained immunity may provide a “head start” to fight novel pathogens that attenuate transmission, progression, or severity of infection. For instance, a plethora of evidence on the protective role of BCG resulted in at least 25 clinical trials in the early stage of the COVID-19 pandemic [20].

**Figure 3:**
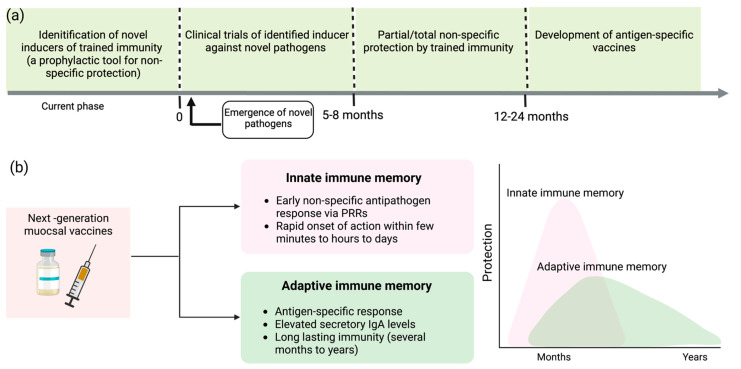
Bolstering trained immunity to elicit rapid response against emerging respiratory infections and to augment the efficacy of conventional vaccines. (**a**) Identification of safe and efficient inducers of trained immunity facilitates an innovative approach to fight or prevent the progression of novel pathogens. This provides valuable time to develop antigen-specific vaccines against novel microbes. (**b**) Next-generation hybrid vaccines integrate the unique features of innate and adaptive immune memory that can lead to a rapid onset of action and enhance the longevity of the protection window.

Identification of more biocompatible and safer inducers of trained immunity may facilitate repeated dosing and increase the probability of improving the innate response to amplify protection against new pathogens. Foreseeing the threat of unavoidable outbreaks, preclinical studies and clinical trials should be designed to assess short-term outcomes. The prospect of achieving more robust protection against respiratory pathogens is higher with intranasal than parental administration. For instance, nasal administration of the BCG vaccine showed 100% protection against a deadly strain of influenza virus that invades the upper respiratory tract [51]. Hence, the strategical development of novel adjuvants that boost trained immunity could be a new paradigm for prophylaxis against emerging respiratory infections, the leading cause of unanticipated epidemics and pandemics, as illustrated in Figure 3a.

## 6. Integrating Innate and Adaptive Immune Memory in New-Generation Vaccines

BCG, a paradigm inducer of trained immunity, amplifies the magnitude and robustness of the innate immune response. However, the complications of BCG in a cohort of immunosenescent and immunocompromised individuals restrict its widespread use in a diverse population. Moreover, the hallmark of trained immunity, upregulated cytokine secretion [67], may increase the risk of autoimmunity. Although no such detrimental outcome has been reported for BCG vaccines, it is a critical attribute to examine in preclinical and clinical studies, and the necessity of safety evaluations is high for novel trained immunity inducers. Comprehensively studied novel adjuvants may surpass the limitations of BCG for repeated administration, the longevity of immune response, and ubiquitous utility for diverse populations.

Through this review, I concur with the notion of integrating novel adjuvants/immunity inducers with antigen-specific vaccines to devise new-generation vaccines that can synergistically produce a durable immune response, as proposed by Netea et al. [67]. The hybrid approach to novel vaccine development incorporates the memory traits of both innate and adaptive immunity, facilitating a rapid, non-specific immune response against unidentified pathogens and antigen-specific immune exclusion of microbes, as mediated by adaptive immunity [67] (Figure 3b). Therefore, hybrid vaccines that increase the efficacy of conventional vaccines represent a promising preventive approach for fighting respiratory infections.

## 7. Conclusions

Pandemics instigated by respiratory pathogens significantly contribute to the health crisis. In the light of emerging COVID-19 variants, improving vaccine efficacy is of utmost importance. Trained immunity holds a tremendous potential to elicit cross-reactivity to heterologous infections, unrealizable in classic vaccines. The review discusses on leveraging trained immunity to combat emerging pathogens and improving the efficacy of conventional vaccines. Novel inducers of trained immunity need to be identified for safer and repeated administration to enhance the longevity of protection. Despite the existing knowledge on trained immunity, extensive investigation is required to comprehensively understand the epigenetic and metabolic pathways and determine the range of cells that contribute to innate immune memory. In addition, the function of noncoding RNAs that modulate innate immune genes warrants further research. Cutting-edge tools, including single-cell sequencing, could be utilized to explore subsets of the immune population that have demonstrated innate immune responses. In conclusion, trained immunity is an indispensable defensive tool to forestall pathogen transmission, disease severity, and mortality in future pandemics and could also be employed in tandem with antigen-specific vaccines to confer complete protection against emerging variants.

## Data Availability

Not applicable.

## Abbreviations

PPRs—pathogen recognition receptors, GM-CSF—granulocyte-macrophage colony-stimulating factor, BCG—Bacille Calmette-Guerin, NK—natural killer cells, RSV—Respiratory Syncytial Virus, LPS—Lipopolysaccharides, HSC—hematopoietic stem cells, PFU—Plaque Forming Units, TB—tuberculosis, MPP—multipotent progenitor, GMP—granulocyte-macrophage progenitor, PAMPs—pathogen-associated molecular patterns.
